# MELK-T1, a small-molecule inhibitor of protein kinase MELK, decreases DNA-damage tolerance in proliferating cancer cells

**DOI:** 10.1042/BSR20150194

**Published:** 2015-11-13

**Authors:** Lijs Beke, Cenk Kig, Joannes T. M. Linders, Shannah Boens, An Boeckx, Erika van Heerde, Marc Parade, An De Bondt, Ilse Van den Wyngaert, Tarig Bashir, Souichi Ogata, Lieven Meerpoel, Aleyde Van Eynde, Christopher N. Johnson, Monique Beullens, Dirk Brehmer, Mathieu Bollen

**Affiliations:** * Oncology Discovery, Janssen Research & Development, a division of Janssen Pharmaceutica NV, Turnhoutseweg 30, 2340 Beerse, Belgium; † Laboratory of Biosignaling & Therapeutics, KULeuven Department of Cellular and Molecular Medicine, University of Leuven, Campus Gasthuisberg, O&N1/ Box 901, Herestraat 49, 3000 Leuven, Belgium; ‡ Computational Sciences, Janssen Research & Development, a division of Janssen Pharmaceutica NV, Turnhoutseweg 30, 2340 Beerse, Belgium; § Astex Pharmaceuticals, 436 Cambridge Science Park, Milton Road, Cambridge CB4 0QA, U.K.

**Keywords:** chemical biology, deoxyribonucleic acid (DNA) damage response, maternal embryonic leucine zipper kinase (MELK) kinase, senescence, small molecule inhibitors

## Abstract

Maternal embryonic leucine zipper kinase (MELK), a serine/threonine protein kinase, has oncogenic properties and is overexpressed in many cancer cells. The oncogenic function of MELK is attributed to its capacity to disable critical cell-cycle checkpoints and reduce replication stress. Most functional studies have relied on the use of siRNA/shRNA-mediated gene silencing. In the present study, we have explored the biological function of MELK using MELK-T1, a novel and selective small-molecule inhibitor. Strikingly, MELK-T1 triggered a rapid and proteasome-dependent degradation of the MELK protein. Treatment of MCF-7 (Michigan Cancer Foundation-7) breast adenocarcinoma cells with MELK-T1 induced the accumulation of stalled replication forks and double-strand breaks that culminated in a replicative senescence phenotype. This phenotype correlated with a rapid and long-lasting ataxia telangiectasia-mutated (ATM) activation and phosphorylation of checkpoint kinase 2 (CHK2). Furthermore, MELK-T1 induced a strong phosphorylation of p53 (cellular tumour antigen p53), a prolonged up-regulation of p21 (cyclin-dependent kinase inhibitor 1) and a down-regulation of FOXM1 (Forkhead Box M1) target genes. Our data indicate that MELK is a key stimulator of proliferation by its ability to increase the threshold for DNA-damage tolerance (DDT). Thus, targeting MELK by the inhibition of both its catalytic activity and its protein stability might sensitize tumours to DNA-damaging agents or radiation therapy by lowering the DNA-damage threshold.

## Abstract (precis)

Protein kinase MELK has oncogenic properties and is highly overexpressed in some tumors. In the present study, we show that a novel MELK inhibitor causes both the inhibition and degradation of MELK, culminating in replication stress and a senescence phenotype.

## INTRODUCTION

The preservation of genomic integrity is essential for cell homoeostasis. Aberrant DNA structures can arise during an unperturbed S-phase [1]. If the DNA polymerase encounters an anomalous DNA structure, it will stall. Prolonged stalling of replication forks leads to fork collapse, formation of DNA double-strand breaks (DSBs) and ultimately genome instability. However, DNA-damage tolerance (DDT) pathways have evolved to allow replication through and beyond an altered template [2]. In addition, a well-co-ordinated network of signalling cascades, termed the DNA-damage response (DDR), is activated. The DDR senses DNA aberrations and transmits the damage signal to activate cell-cycle checkpoints in order to delay cell cycle progression and allow more time for repair. In the event of irreparable DNA damage, the damaged cells will be eliminated from the proliferative pool by either induction of senescence or cell death [3].

Cancer cells show multiple abnormalities, including inherent replication stress and DNA damage. They are able to survive and proliferate because key checkpoints are weakened, thereby increasing the barrier for activation of DDR signalling [4]. As a result, cancer cells undergo cell division in an uncontrolled way. Conventional chemotherapy seeks to kill cancer cells by causing further DNA damage, which is sufficient to reach the increased threshold for DDT. Alternatively, a therapeutic agent that could reactivate a checkpoint has the potential to re-enable the cell to detect and respond to DNA damage by senescence or cell death.

Maternal embryonic leucine zipper kinase (MELK) belongs to the sub-family of AMP-activated serine/threonine protein kinases. It is the only member that does not require the liver kinase B1 (LKB1) kinase for its activation [5]. Instead, it is activated through autophosphorylation induced by a currently unknown trigger [6] and potentially through phosphorylation by cyclin-dependent kinases (CDKs) and mitogen-activated protein (MAP) kinases [7]. MELK plays a major role in various cellular and biological processes including neural progenitor cell renewal [8], apoptosis [9,10], mRNA splicing [11], DNA repair [12] and asymmetric cell division [13].

MELK has emerged as a potentially important target for cancer therapy. Indeed, MELK expression is dramatically increased in cancers of various tissue origins with a high proliferation index, including glioblastomas that are known to be resistant to chemo- and radiotherapy [14–16]. Moreover, a direct correlation between high MELK expression and malignancy grade has been reported in solid tumours like melanoma [17], breast cancer [18], brain tumours [15,16] and, also recently, in haematological malignancies like acute myeloid leukaemia (AML) [19]. The high expression level of MELK in undifferentiated cancer cells suggests a possible role for MELK in cancer stem-cell maintenance and survival [16]. Tumour cells may derive a proliferative advantage from the ability of MELK to inhibit p53 (cellular tumour antigen p53) [20] and induce apoptosis [9,10]. In addition, MELK has been implicated in DDR pathways [12,21,22] and in the resistance of tumour cells to DNA-damaging treatments [22]. Importantly, normal differentiated cells have a very low expression level of MELK, offering the promise of a manageable therapeutic window due to absent or low on-target toxicity.

Previously, the novel quinolone-based compound OTSSP167 was reported to inhibit MELK kinase activity [23]. OTSSP167 was also shown to decrease solid-tumour growth from cancer cell lines with high MELK levels, as deduced from xenograft assays. The demonstrated *in vitro* and *in vivo* efficacy and safety in pre-clinical studies has led to the initiation of a clinical trial phase-I program for OTSSP167. We have recently described the tailor-made development of a potent (IC_50_=37 nM) and cell-permeable small molecule inhibitor of the kinase domain of MELK, coined MELK-T1 (Figure 1A) [24]. In the present study, we show that MELK-T1 not only inhibits MELK but also triggers its proteasome-mediated degradation. Moreover, we demonstrate that MELK-T1 induces replication stress and activates the immediate and delayed ataxia telangiectasia mutated (ATM)-mediated DDRs that eventually lead to a replicative senescent phenotype.

**Figure 1:**
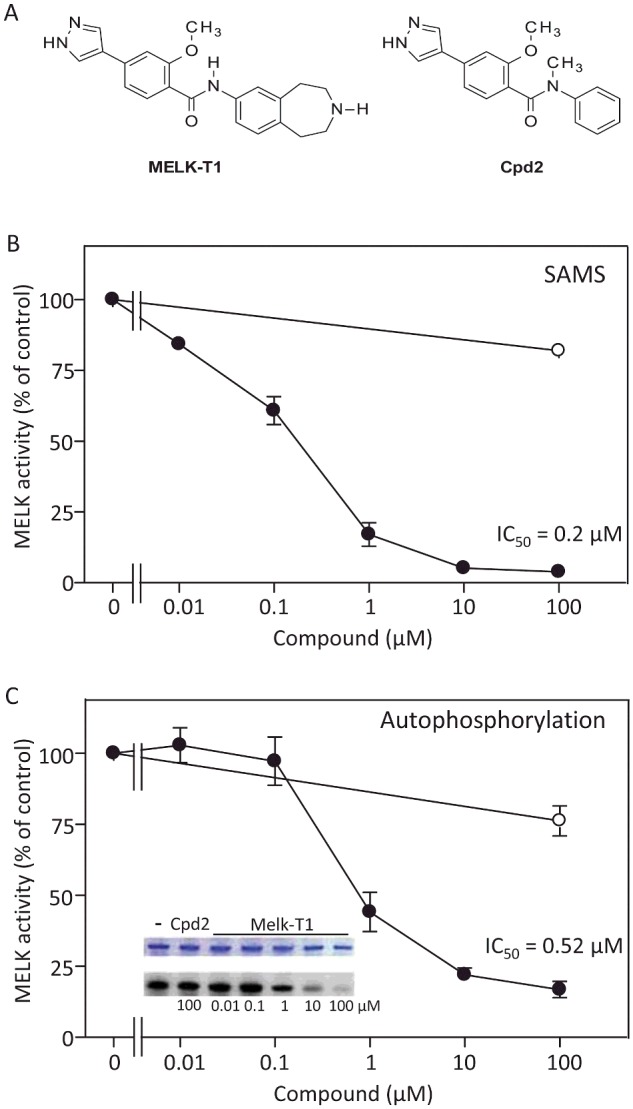
Inhibition of MELK by MELK-T1 (**A**) Structures of MELK-T1 and Cpd2. (**B**) Dose-response curves of MELK-T1 (●) and Cpd2 (○) for the inhibition of EGFP–MELK in an *in vitro* kinase assay with SAMS peptide as substrate. The results from three independent experiments were expressed as mean ± S.E.M. (**C**) Inhibition of MELK autophosphorylation by MELK-T1. Dose-response curves of MELK-T1 (●) and Cpd2 (○) were calculated by measuring the corresponding band intensities obtained by autoradiography (inset shows representative autoradiogram and corresponding Coomassie stained gel). Band intensity values were normalized against the corresponding bands obtained from Coomassie stained gel. The results are expressed as mean ± S.E.M. (*n*=3).

## EXPERIMENTAL

### Cell culture and analysis

MCF-7 cells were cultured in high-glucose DMEM (Dulbecco's modified Eagle's medium; LifeTechnologies), supplemented with 10% FBS (LifeTechnologies). Transfection of EGFP-MELK and knockdown of MELK with siRNA were performed as described previously [25]. A549 lung adenocarcinoma cells were cultured in DMEM (Life Sciences) supplemented with 10% FCS (fetal calf serum; Thermo Scientific), 2 mM L-glutamine (Life Sciences), 1 mM sodium pyruvate (Life Sciences) and 50 μg/ml gentamicin (Life Sciences).

FACS analyses [25] and i*n vitro* IC_50_ data profiling were carried out as described [24]. *IncuCyte-*Growth curves based on confluency measurements were constructed by imaging plates using the IncuCyte system (Essen Instruments). BrdU (bromodeoxyuridine) incorporation assays and analysis of replication fork dynamics were described previously [25]. In these experiments, the compounds were added 2 h before fixing or harvesting the cells. For EdU (5-ethynyl-2′-deoxyuridine) incorporation assays, the Click-iT® Plus EdU Alexa Fluor® 488 Imaging kit (C10637) of LifeTechnologies was used according to the manufacturer's instructions. Images were scanned with the In Cell Analyzer 2000 (GE Healthcare Life Sciences), objective Nikon 10×0.45 and the following filters: DAPI (exposure 25 ms) and FITC (exposure: 200 ms).

For 3D-sandwich assays, MCF-7 cells were pre-treated with the indicated compounds for 72 h in a 2D-setting before being transferred to a BME (basement membrane extract)-gel matrix. The pre-treated cells were embedded between two layers of Trevigen Cultrex BME (Trevigen 3433-005-01) as follows. First, a 24-well plate was coated with ice-cold undiluted BME and incubated for 1 h at 37°C to solidify. Next, the cells were plated on the first BME gel layer and incubated for 3–4 h to allow attachment. After aspiration of the medium, the cells were covered with ice-cold BME matrix, diluted with culture medium (BME medium=1:1). The plate was again incubated for 1 h to allow the second BME layer to solidify. Finally, culture medium supplemented with the compound was added on top of the second BME matrix. The assay plate was placed in a Cell-IQ imager (Chipman Technologies), equipped with 5% CO_2_ supply and images were taken every 2 h for the next 10 days.

### Antibodies

The following antibodies were used: Actin (Sigma), ATM (D2E2, Cell Signaling Technologies), P-ATM (Ser1981, Abcam), CHK2 (checkpoint kinase 2; Cell Signaling Technologies), P-CHK2 (Thr68, Cell Signaling Technologies), MELK (Sigma), p21 (cyclin-dependent kinase inhibitor 1; Upstate), p53 (PAB 1801, Santa Cruz), p53 DO-1 (Santa Cruz), P-p53 (Ser15, Cell Signaling Technologies) and Vimentin (Santa Cruz).

BrdU and γH2A.X (phospho-histone H2AX) immunostainings were performed following a treatment for 2 h with the indicated compounds. Cells were fixed in 10% formalin solution (Sigma–Aldrich) for 10 min at room temperature and 20 min in ice-cold methanol, after which they were washed 3× in PBS. Subsequently, the cells were blocked for 1 h in PBSAT (PBS+1% BSA+0.1% Tween) and incubated overnight with the primary antibody in PBSAT. The cells were washed 3× with PBSAT and incubated with the secondary antibody for 1 h at room temperature, again washed 3× with PBSAT and incubated with the Hoechst staining solution (1/2000 in PBSAT).

### *In vitro* kinase assays

MCF-7 cells were transiently transfected with EGFP–MELK and lysed after 48 h, as previously described [6]. EGFP–MELK was immunoprecipitated using GFP-trap beads (Chromotek). The bead suspension was used in an *in vitro* kinase assay with the indicated concentrations of the compounds for autophosphorylation of MELK or with SAMS (substrate peptide for 5′-adenosine monophosphate-activated protein kinases (AMPKs) peptide. Autophosphorylation of MELK was analysed by autoradiography after SDS/PAGE. The IC50 values were calculated from the dose-response curves via non-linear regression curve fit in GraphPad Prism.

### Microarrays

MCF-7 cells were treated with 10 μM MELK-T1 for 48 h. Cells were lysed using RLT (RNeasy lysis) buffer (Qiagen) and RNA was extracted with the RNeasy 96 kit (Qiagen). All microarray-related steps for target preparation, including the amplification of total RNA and labelling, were carried out as described in the GeneChip®3′ IVT Express Kit User Manual (Affymetrix 2004). Biotin-labelled target samples were hybridized to GeneChip® Human Genome HT U133 PLUS PM 96-Array containing probes for approximately 19000 genes. Target hybridization was processed on the GeneTitan® Instrument according to the instructions provided in the User Guide for Expression Array Plates. Images were analysed using the GeneChip® Command Console Software (AGCC; Affymetrix). All data were processed using the statistical computing R-program (R version 3.0.1; R Development Core Team) and Bioconductor tools [26]. The gene expression values were computed using RMA (robust multi-array average) [27]. Grouping of the individual probes into gene-specific probe sets was performed based on Entrez Gene using the metadata package hthgu133pluspmhsentrezg (version 15.1.0 [28]). The resulting log_2_-transformed data were the basis for differential expression analysis using Significance Analysis of Microarrays software [29]. The affected pathways were analysed using MLP (mean log P analysis) [30] and IPA (Ingenuity Pathway Analysis; Ingenuity Systems, www.ingenuity.com). The data have been submitted in GEO (gene expression omnibus) (GSE62477).

### Statistics

The results are presented as means ± S.E.M. for at least three independent experiments. Statistical significance between the control and the experimental groups was tested using Student's *t* test.

## RESULTS

### Inhibitory potency of MELK-T1

As described previously [24], the transamide carbonyl oxygen of MELK-T1 (Figure 1A) binds to the backbone NH of Cys89 in the hinge region of the catalytic domain of MELK. In addition, the *N*-phenyl benzamide core is kept in a planar conformation by an intramolecular hydrogen bond between the ortho-methoxy and the amide-NH groups. We synthesized an analogue of MELK-T1 named Compound 2 (Cpd2) (Janssen R&D; Figure 1A), in which the methylation of the amide disturbs the planarity of the *N*-phenyl benzamide and probably also the binding to the hinge region. Accordingly, this compound showed no detectable inhibition of MELK activity *in vitro* (IC_50_ > 10 μM) and was used as a negative control. To characterize the potency of MELK-T1 against full-length MELK protein, exogenously expressed EGFP–MELK was immunoprecipitated from MCF-7 cells. Next, its activity was measured using the SAMS peptide as substrate in the presence of the indicated concentrations of the compounds (Figure 1B). An IC_50_ of 0.2 μM was determined for MELK-T1, but no inhibitory activity could be detected for Cpd2. Similar data were obtained when the kinase activity was derived from the autophosphorylation of MELK (IC_50_=0.52 μM) (Figure 1C). These IC_50_ values for full-length MELK were 5–14-fold higher than previously reported for the catalytic domain (residues 1–340) of MELK [24], indicating that the C-terminal regulatory domain and/or interacting proteins somehow decrease the affinity for MELK-T1.

### MELK-T1 triggers the degradation of endogenous MELK

To compare the effects of MELK-T1 and Cpd2 on cultured MCF7 cells, we used the compounds at a final concentration of 10 μM. The required concentration was higher than that needed to inhibit purified MELK (Figure 1), hinting at a limited permeability or stability of MELK-T1 in MCF7 cells. It was also higher than the concentration (1.5 μM) previously used for Ba/F3 cells [24], indicating that the sensitivity to MELK-T1 is cell-type dependent.

Exposure of unsynchronized MCF-7 cells (Figure 2A) to MELK-T1 triggered a fast reduction in the endogenous MELK protein level within 4 h of compound addition. The MELK protein level is known to be strictly controlled [31,32]. Indeed, the kinase is expressed in proliferating cells, but is undetectable in differentiated cells. Also, the MELK-encoding gene is an E2F (transcription factor E2F1) target and is induced during S-phase [31]. The protein level reaches a maximum during the G2/M-phase of the cell cycle and drops again at the mitotic exit [31]. The fast reduction in endogenous MELK protein after the addition of MELK-T1, irrespective of the cell-cycle phase, hints at a direct effect of the compound on protein stability rather than an effect through interference with cell-cycle progression. Moreover, a similarly fast loss of MELK protein was detected in the A549 lung adenocarcinoma cell line, excluding a cell-line-specific effect (Supplementary Figure S1).

**Figure 2:**
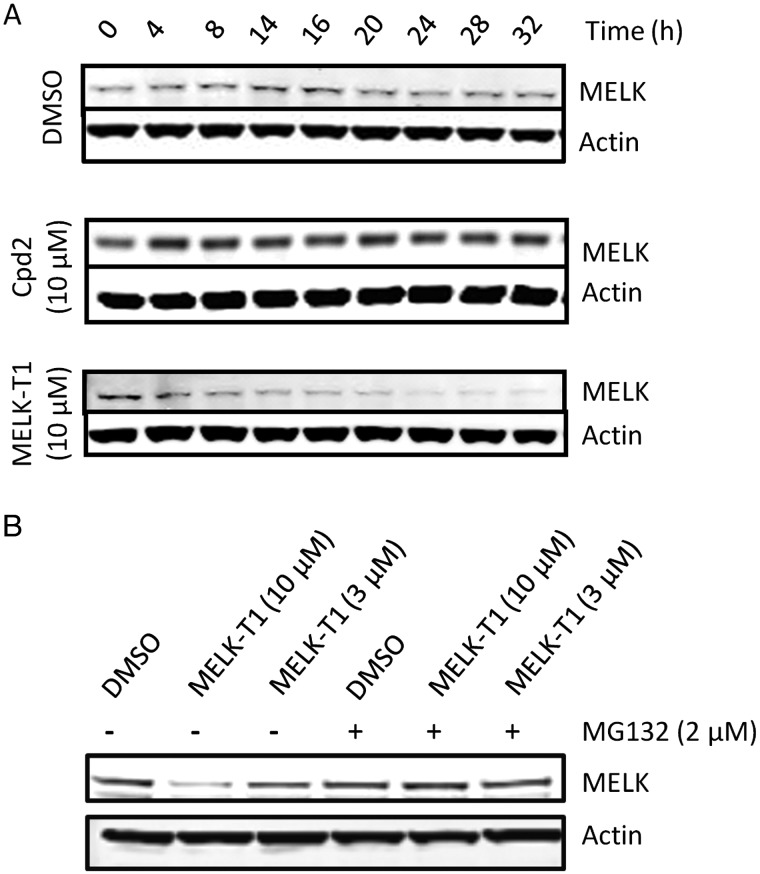
MELK-T1 triggers the proteasome-mediated degradation of MELK protein (**A**) Immunoblot analysis of MELK and actin levels in lysates of compound-treated MCF-7 cells for the indicated times. (**B**) Immunoblot of MELK and actin levels in lysates of MCF-7 cells that were pre-treated with 2 μM of the proteasome inhibitor MG132 for 1 h before addition of the indicated compound for 6 h. The blots are representative for three experiments.

To further explore the MELK-T1-induced down-regulation of MELK, MCF-7 cells were pre-treated with the proteasome inhibitor MG132 for 1 h prior to the addition of MELK-T1. Interestingly, adding MG132 abolished the MELK-T1-induced reduction in cellular MELK protein (Figure 2B), indicating an active and controlled degradation mechanism by the proteasome.

### Inhibition/degradation of MELK causes an S-phase arrest

FACS of cell-cycle distribution of synchronized MELK-T1-treated MCF7 cells revealed a significant reduction in the percentage of S-phase cells, with a corresponding increase in the G1 cell population (Figure 3A). This observation is in agreement with our previous work showing a delay in S-phase progression after a MELK knockdown in U87 glioblastoma cells [25]. Next, MCF7 cells were grown in the presence of EdU, a nucleoside analogue of thymidine that is incorporated into DNA during replication. In accordance with the FACS data, the relative number of EdU-positive cells was decreased after the inhibition of MELK (Figure 3B), consistent with the notion that MELK plays a crucial role in S-phase.

**Figure 3:**
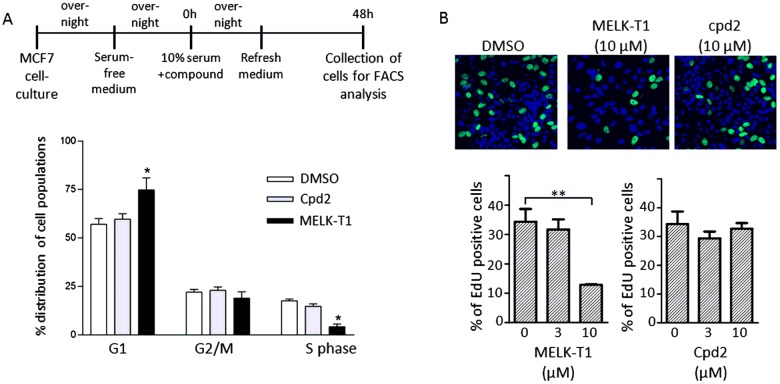
MELK-T1 induces a delay in the progression of MCF-7 cells through S-phase (**A**) Time-line showing the treatments and the incubation periods used for MCF-7 cell cultures. The histograms show the cell-phase distribution of MCF-7 cells that were treated as explained in the time-line. Results are expressed as percentage changes (mean ± S.E.M.; *n*=3), *P<0.05. (**B**) Representative immunohistochemistry images of MCF-7 cells treated with the indicated compounds for 96 h showing EdU incorporation (green) and Hoechst staining (blue). The percentage of positively stained EdU cells was normalized to the percentage of positively stained EdU cells of the DMSO control condition (0 μM). The results are expressed as mean ± S.D. (*n*=3), **P<0.01.

### The down-regulation of MELK induces stalled replication forks and DSBs

To exclude general DNA-damaging properties of MELK-T1, we performed both DNA mobility shift studies (Figure 4A) and DNA intercalation studies (Figure 4B). At concentrations up to 50–100 μM MELK-T1 did not show direct DNA damaging properties in these *in vitro* assays.

**Figure 4:**
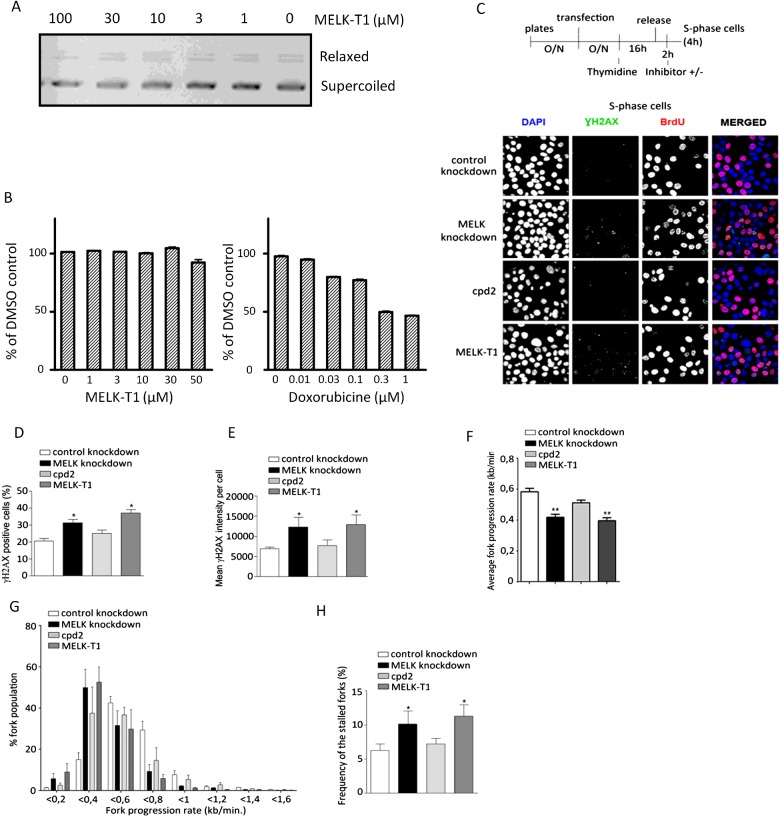
Effects of MELK-T1 on DNA structure and replication (**A**) The agarose gel shows a DNA-mobility shift assay performed with the indicated concentrations of MELK-T1. (**B**) An *in vitro* DNA intercalation assay was performed taking doxorubicin, a known DNA intercalator, as a positive control. Fluorescence measurements of an ethidium bromide competition assay were performed with a concentration range of MELK-T1 and doxorubicin. The results (percentage of DMSO control) are expressed as mean ± S.D. (*n*=3). (**C**) Schematic of the procedure used to synchronize MCF-7 cells in S-phase. The cells were incubated with BrdU (30 μg/ml) for 45 min before fixation. O/N, overnight. Representative images from confocal microscopy showing γH2A.X, BrdU and DAPI staining in cells treated as illustrated in the time-line. (**D**) Quantification of the number of γH2A.X positive cells (percentage of total). At least 300 cells were counted in each condition. Cells with five or more foci were considered γH2A.X positive. The results are expressed as mean ± S.E.M. (*n*=3). *P<0.05. (**E**) Comparison of the average γH2A.X raw signal intensity from at least 500 cells for the indicated conditions. The results from three independent experiments were expressed as mean ± S.E.M. *P<0.05. (**F**) Fork-progression rate was quantified as described previously [25] under the indicated conditions and expressed as kb/min. At least 500 fibres were counted from each condition in three independent experiments. The results are expressed as mean ± S.E.M. **P<0.001. (**G**) Distribution of the fork rates among the fibre populations in the indicated conditions. The results are expressed as mean ± S.E.M. (*n*=3). (**H**) Quantification of stalled forks (ratio of stalled forks/number of ongoing forks × 100). At least 70 replication units were counted for each condition in three independent experiments. The results are expressed as mean ± S.E.M. *P<0.05.

Next, we explored whether the down-regulation of protein caused replication stress in MCF-7 cells. Both the siRNA-mediated knockdown of MELK and a short-term treatment with MELK-T1 (2 h) resulted in an increase in DSBs in MCF-7 cells synchronized in S-phase (Figures 4C–4E), as visualized by the accumulation of γH2A.X foci. The down-regulation of MELK also caused a clear increase in the number of cells with γH2A.X foci in asynchronized cells, but had no such effect in cells that were arrested by serum starvation in G_0_ (Supplementary Figure S2). In these experiments, the simultaneous staining for BrdU incorporation confirmed that the increased accumulation of γH2A.X was only detected in cells undergoing DNA replication. Collectively, these data clearly show that the induction of DSBs is S-phase specific.

Subsequently, the replication in MCF-7 cells was monitored by the successive labelling of newly synthesized DNA with the thymidine analogues iododeoxyuridine (IdU) and chlorodeoxyuridine (CldU). This allows the measurement of fork progression and the quantification of stalled forks [25]. Inhibition or knockdown of MELK resulted in a 35% decrease in the average length of fibres labelled with CldU, reflecting an average decreased fork progression speed (Figure 4F). A representation of the distribution of frequency of fork velocities (Figure 4G) showed that both inhibition and knockdown of MELK caused a shift, with a reduced number of fast-progressing forks and an increased number of slowly-progressing forks. Moreover, the inhibition or knockdown of MELK was associated with an increased number of asymmetrical forks, pointing to a higher frequency (~40%) of stalled forks (Figure 4H). This observation cannot be explained by mechanically-broken DNA fibres, because only forks with the two halogenated labels were considered for the quantitative assays. In conclusion, both the inhibition and the depletion of MELK induced replication stress during an unperturbed S-phase in MCF-7 cells.

### MELK-T1 activates the ATM-mediated DDR

The siRNA-mediated knockdown of MELK leads to activation of the ATM–CHK2 pathway [25]. ATM-mediated downstream signalling in response to DSBs can be divided into two major cascades [33]. During the rapid response, activated ATM phosphorylates and activates the effector kinase CHK2. Activated CHK2 in turn phosphorylates CDC25A (dual specificity phosphatase Cdc25A) and targets it for ubiquitination and degradation. The depletion of CDC25A leads to an accumulation of phosphorylated CDK2–cyclin complexes and therefore a cell-cycle arrest. The delayed response transforms this cell-cycle arrest into a long-term arrest by consecutive activation of ATM and p53, leading to an increased expression of the CDK inhibitor p21.

Both ATM-mediated cascades were monitored in a time-course analysis of MELK-T1-treated MCF-7 cells. ATM and CHK2 were already activated 20 min after the addition of MELK-T1, whereas the sustained p53 activation and p21 up-regulation appeared later (Figures 5A and 5B). Importantly, MELK-T1 triggered the same signalling cascade in a lung-adenocarcinoma cell line A549, indicating that this response is not limited to MCF-7 cells (Supplementary Figures S3A and S3B). Overall, our results indicate that the inhibition and degradation of MELK protein induces both the early and the delayed ATM-mediated DDR response, leading to the induction of a cell-cycle arrest.

**Figure 5:**
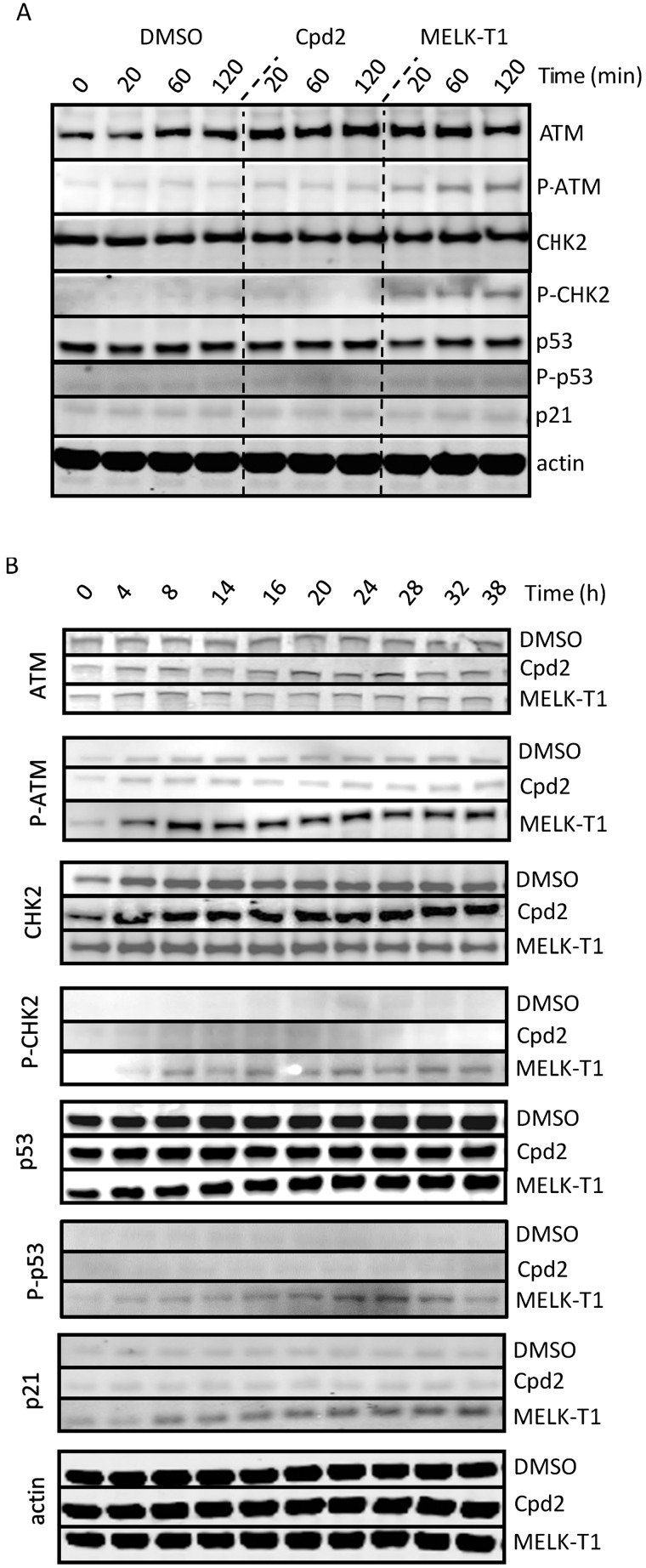
MELK inhibition induces an early and delayed ATM-mediated DDR response Immunoblot analysis with the indicated antibodies of lysates from compound-treated MCF-7 cells for a short period (**A**) or a long period (**B**). P-ATM=ATM phosphorylated on Ser1981, P-CHK2=CHK2 phosphorylated on Thr68, P-p53=p53 phosphorylated on Ser15. The blots are representative for three experiments.

### Transcriptional effects of MELK-T1

To explore whether the effect of MELK inhibition on the ATM-mediated DDR also leads to downstream transcriptional effects, we carried out a gene expression profiling study. A 48-h treatment of MCF-7 cells with 10 μM MELK-T1 resulted in the down-regulation of 648 genes and up-regulation of 607 genes, with a ≥ 1.5-fold change in the transcript level (Supplementary Table S1). IPA revealed that DDR, cell-cycle control of chromosomal replication and ATM signalling were among the most affected pathways (Figure 6A). Approximately 30% of the genes annotated to these pathways were affected, mostly down-regulated, after a treatment with MELK-T1 (Figures 6A and 6B). These data independently confirm that the down-regulation of MELK leads to a global ATM-mediated DDR response.

**Figure 6:**
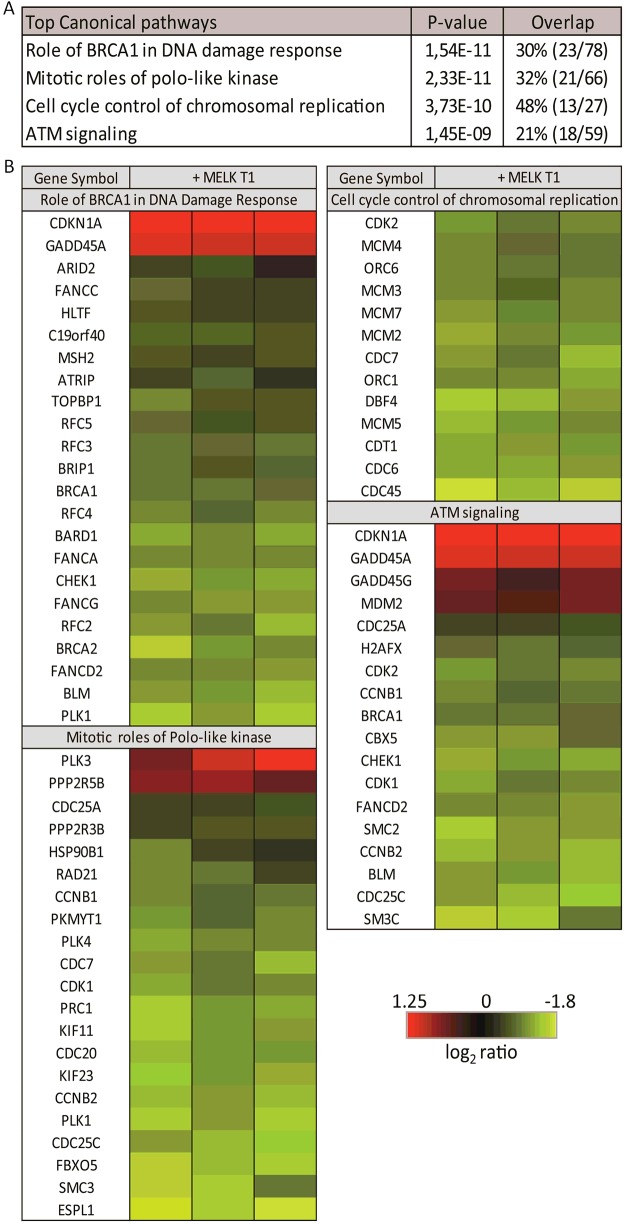
MELK function modulates the expression of genes involved in DNA-damage signalling (**A**) Table representing IPA analysis results of the top canonical pathways affected 48 h after MELK-T1 treatment. ‘Overlap’ demonstrates the percentage of differentially expressed genes in each pathway. (**B**) Heat-map representation of the significant differentially expressed genes in the top canonical pathways as shown in (**A**).

Intriguingly, we also noticed a 2.6-fold down-regulation of FOXM1, an oncogenic transcription factor that forms a complex with MELK and is dependent on phosphorylation by MELK for its activity in glioma stem cells [34]. Recently, Alachkar et al. [19] also noticed a down-regulation of FOXM1 and its downstream target genes upon treatment of AML cell lines with OTSSP167 [19]. Our microrarray gene MELK target list contained 20 FOXM1 target genes and 18 of these were down-regulated (Supplementary Table S2). These included protein kinases PLK1 (Polo-like kinase 1) and PLK4 (Polo-like kinase 4) and explain why we found the mitotic roles of polo-like kinase among the top canonical pathways analysis (Figure 6A). Strikingly, we found that the MELK transcript itself was also significantly down-regulated (2.21-fold). This agrees with the recent report that MELK itself is a FOXM1 target [35].

### A prolonged treatment with MELK-T1 causes growth inhibition and senescence

As expected from the induction of the DDR response and subsequent induction of p21, a prolonged treatment of MCF-7 cells with MELK-T1 caused a stagnant growth at 30%–40% confluency (Figure 7A), as confirmed by a lower number of cells (Figure 7B). Also, this growth arrest was accompanied by an enlarged and flattened cell morphology, which are typical features of senescent cells under 2D-growth conditions (Figures 7A and 7B). MCF-7 cells form distinctive and highly migrating spheroids under specific 3D-sandwich assay conditions. Remarkably, MELK-T1 pre-treated cells lost this characteristic spheroid morphology as well as the capacity to migrate and proliferate (Figure 7C; Supplementary Movies S1–S3), indicating that MELK-T1 seriously reduces cell growth of MCF-7 cells.

**Figure 7:**
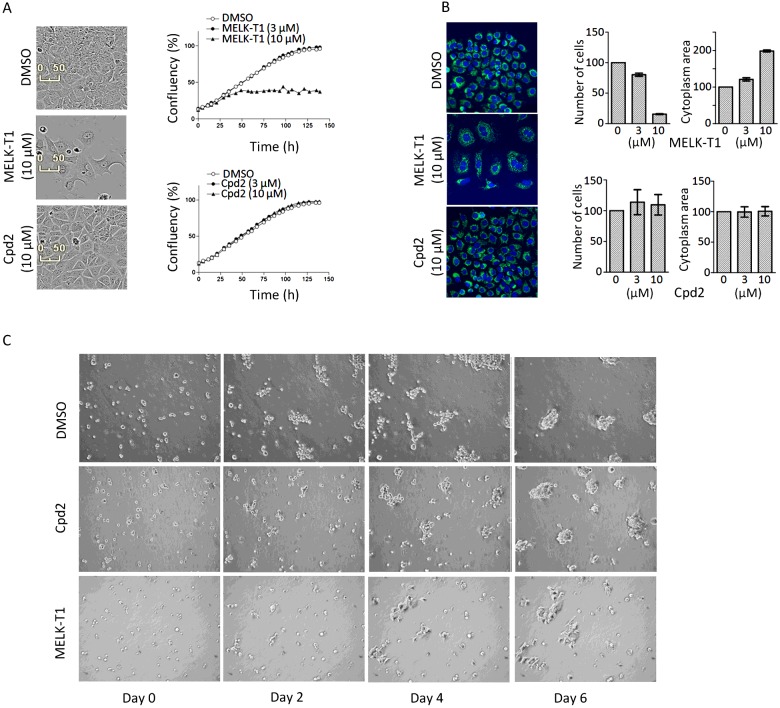
MELK-T1 induces a growth arrest and a senescent phenotype (**A**) Representative phase-contrast images obtained by live content imaging of compound-treated MCF-7 cells over a time course of 140 h and growth curves based on confluency measurements of the phase-contrast images. (**B**) Representative immunohistochemistry images of compound-treated MCF-7 cells for 96 h showing vimentin (green) and Hoechst staining (blue). The left graph shows the number of cells based on nuclear count of the obtained immunohistochemistry images and normalized to the DMSO control (0 μM). In the right graph, the average cytoplasmic area was quantified and normalized to the DMSO control (0 μM). The results are expressed as mean ± S.D. (*n*=3). (**C**) Representative phase-contrast images of a sandwich assay of compound-pre-treated MCF-7 cells followed over a time period of 8 days.

To explore whether the inhibitory effect of MELK-T1 on cell growth is dependent on the genetic background, MELK-T1 was probed against a panel of 81 solid tumour cancer cell lines (© Oncolead GmbH & Co. KG). Statistical analysis was performed using proprietary software developed at Oncolead. In 46 of these 81 cell lines, a GI_50_, i.e. the concentration producing 50% of maximal inhibition of cell proliferation, was reached at a concentration below 20 μM after 96 h of incubation. A representation of the Z-scores is shown in Figure 8. The inhibition of growth by MELK-T1 was not correlated with the genetic background, implying that no molecular biomarker profile could be determined. These data clearly show that MELK has a role in a general oncogenic pathway not linked to a particular cancer type or mutation profile.

**Figure 8:**
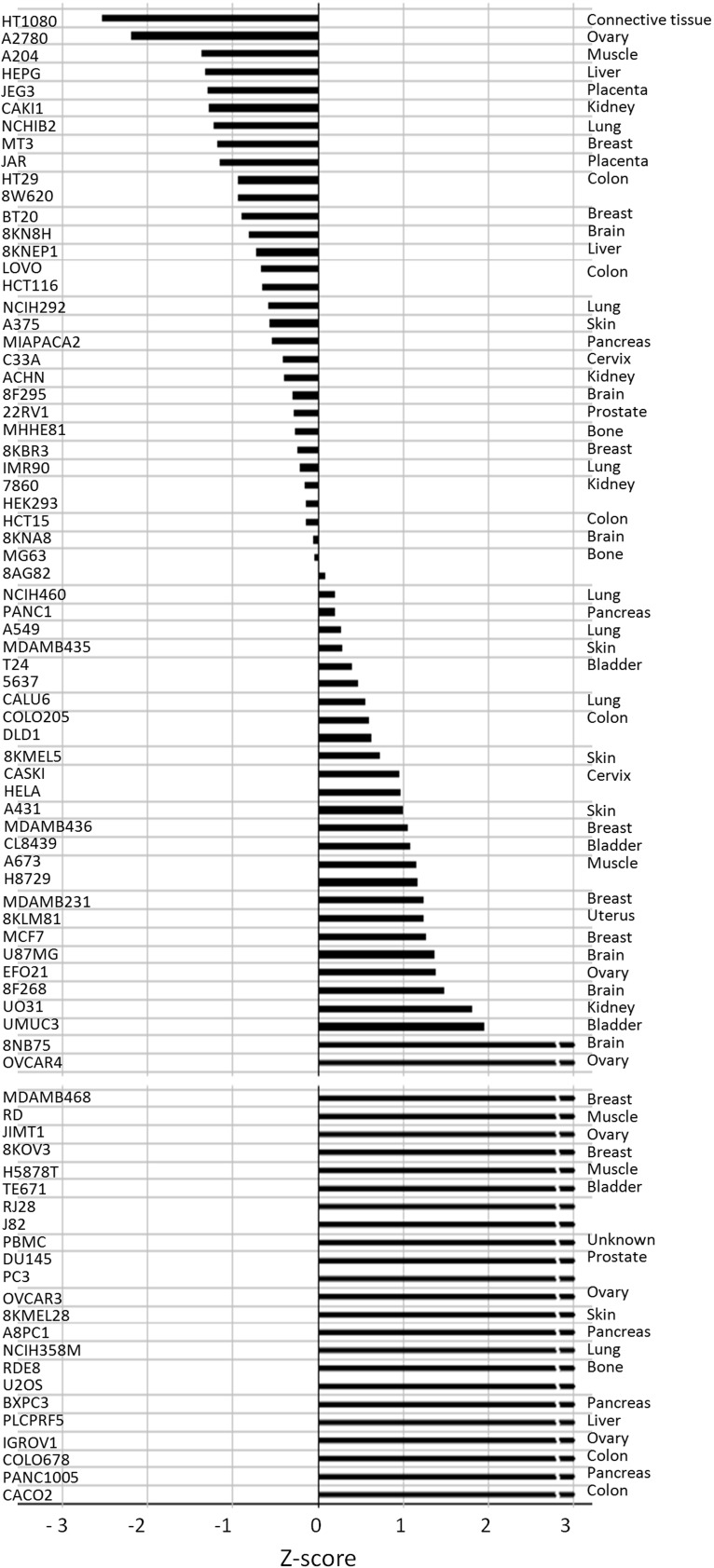
MELK-T1 shows a broad activity range in the Oncolead cell-line panel Z-scores of the incubation with MELK-T1 for 96 h are represented for the indicated cell lines (left) and their tissue origin (right).

## DISCUSSION

So far, only a limited number of MELK inhibitors have been described [36]. These inhibitors are clearly inhibitory to cancer-cell growth, both *in vitro* and in xenograft mouse models, although the underlying molecular mechanism is poorly understood. Therefore, the need for a thorough cellular validation of MELK inhibitors remains high. In the present study, we have applied our recently described inhibitor MELK-T1 to further unravel the biological function of MELK [24].

We observed that MELK-T1 initiates a rapid down-regulation of endogenous MELK by triggering its proteasome-dependent degradation. This effect on MELK protein stability has not been described for any other MELK inhibitor. Since MELK-T1 is a type I, ATP-mimetic inhibitor [24], its binding to MELK possibly stabilizes the ATP-bound conformation, which could trigger the degradation of MELK protein by either of two mechanisms. Firstly, MELK is a known Hsp90 (heat shock protein 90) client [37]. Protein kinase clients are recruited to the Hsp90 molecular chaperone system via Cdc37 (Hsp90 co-chaperone Cdc37), which simultaneously binds Hsp90 and a kinase and regulates the Hsp90 chaperone cycle. However, ATP-competitive inhibitors such as MELK-T1 can deprive the kinases of access to the Hsp90–Cdc37 chaperone system, leading to their degradation [38]. Secondly, acquisition of the fully active conformation of MELK, capable of ATP-binding and substrate binding, is controlled at multiple layers. MELK activity is dependent on reducing agents, owing to an intramolecular disulfide bond that leads to structural distortions [39] and also on an autophosphorylation event at Thr^167^ [6]. Moreover, the ubiquitin-associated domain (UBA) located next to the kinase domain of MELK contributes to protein folding and proper conformation of the kinase domain [39]. UBA domains bind to ubiquitin and contribute to all ubiquitin-dependent cellular processes, including proteasome-mediated degradation. A nucleotide-dependent communication between sub-domains can therefore be postulated which could reduce the half-life of the activated (ATP-bound) conformation of the MELK protein. A high affinity ATP-mimetic inhibitor, such as MELK-T1, might stabilize the active conformation and in this way trigger active degradation via ubiquitination.

We have previously shown that a lack of MELK, caused by its siRNA-mediated knockdown in U87 cells, results in a proliferation arrest [25]. A similar phenotype was observed in MELK-T1-treated MCF-7 cells (present work). In addition, we observed a MELK-T1 induced down-regulation of FOXM1 and a corresponding decreased expression of its target genes. This agrees with earlier described effects of the MELK inhibitor OTSSP167 in AML cell lines [23]. A prolonged treatment of MCF-7 cells with MELK-T1 also inhibited MELK transcription, most probably via down-regulation of FOXM1. The lower MELK transcript level combined with an increased MELK protein degradation results in an almost complete elimination of MELK protein which, moreover, potentially induces a feedback loop through a further reduction and inactivation of FOXM1. This long-term shutdown of both MELK and FOXM1 may be a requirement for the observed irreversible senescence phenotype after prolonged incubation with MELK-T1.

Exploring the mechanism of action of MELK-T1 that leads to the observed proliferation arrest, we demonstrated that inhibition and active degradation of MELK protein in MCF-7 and A549 cells activate an ATM-mediated DDR. In particular, MELK inhibition/depletion leads to the consecutive phosphorylation of ATM, CHK2, p53 and the up-regulation of p21, as well as an accumulation of γH2A.X foci in S-phase. Moreover, an increased incidence of stalled replication forks and a prolonged S-phase were observed. Our finding that the knockdown of MELK and the addition of MELK-T1 caused the same type of replication stress is strong evidence that MELK-T1 acts through inhibition of MELK. In this respect, it is also worthy of note that Cpd2, despite its very similar structure, had no such effects. Nevertheless, as is true for any drug, it is difficult to entirely rule out off target effects of MELK-T1.

Normal cells have a low expression level of MELK and effective checkpoints which ensure a balanced equilibrium between DDR and senescence or apoptosis in the event of unrepairable DNA (Figure 9A). In cancer cells, however, MELK is often overexpressed. An increased MELK function may be crucial to overcome DNA-damage checkpoint activation upon replication stress and thereby avoid senescence or apoptosis (Figure 9B). Moreover, higher MELK levels can contribute to an increased resistance to DNA-damage inducing chemotherapeutics or radiation therapy. Inhibition and depletion of MELK protein by MELK-T1 treatment may re-enable cancer cells to detect and respond to DNA damage (Figure 9C).

**Figure 9:**
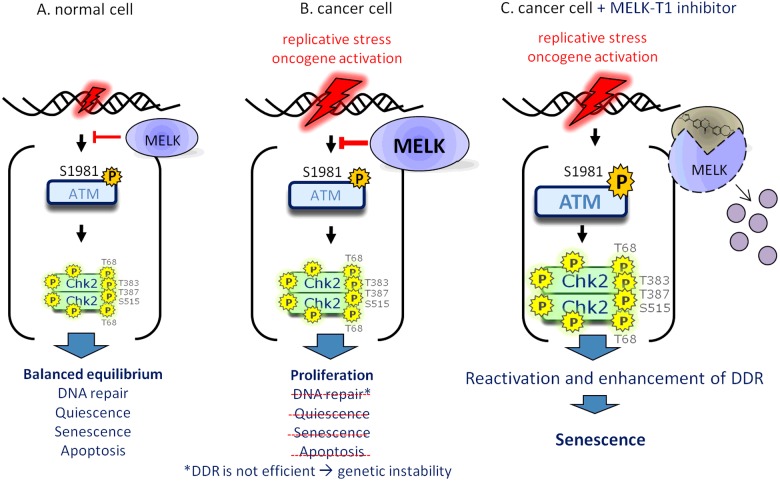
Model of how MELK increases the DDT barrier (**A**) Normal differentiated cells have a low expression level of MELK. In the event of DNA damage, the ATM-mediated DDR is switched on to maintain cell homoeostasis by a balanced equilibrium between repair, in the case of manageable damage and proliferation arrest or cell death, in the case of irreparable DNA damage. (**B**) In cancer cells MELK is overexpressed and keeps the ATM-mediated DDR in check, rendering the cell unable to respond. This co-incides with an accumulation of stalled replication forks, formation of DSBs and therefore genetic instability. (**C**) Inhibition and degradation of MELK by MELK-T1 in cancer cells lowers the threshold for DDT and reactivates/enhances the DDR. This sensitizes cancer cells to their inherent DNA damage and replication stress and results in growth arrest and senescence.

Importantly, an inhibitor-induced degradation of MELK has not been described for any other MELK inhibitor. MELK-T1 could therefore display a different mode of action. Drugs that induce protein degradation are likely to have different pharmacological effects from those that simply obstruct a binding site, as kinase-independent signalling will also be eliminated. The up-regulation of MELK in cancers is due to an increased protein level and not to gain-of-function mutations, as is seen for several other oncogenic kinases [40]. Importantly, both increased MELK activity and protein level contribute to cancer-cell proliferation [36]. Accordingly, we noted that the effects of a knockdown of MELK in glioblastoma cells could be partially rescued with a catalytic-death mutant of MELK [25], hinting at kinase-independent functions of MELK in cell proliferation.

In the present manuscript, we mainly focused on the MCF-7 breast adenocarcinoma cell line. The effective treatment of breast cancer remains a major challenge, owing to the recurrence of treatment-resistant tumours. This includes the use of platinum-based chemotherapy, which fails because tumours acquire tolerance to drug-induced DNA damage. Targeting MELK function by MELK-T1 might therefore sensitize tumours to DNA-damaging agents or radiation therapy by lowering the threshold for DDT. Alternatively, MELK inhibitors as a single-agent therapy could be appropriate for cancers suffering to a sufficiently high extent from inherent replication stress. On the other hand, the growth inhibitory effects of MELK-T1 on a broad panel of cancer cell lines indicate that MELK is an interesting therapeutic target for several cancers and that MELK-T1 is a very promising lead compound due to its combined effect on MELK activity and stability.

## Abbreviations

AML: acute myeloid leukaemia
AMPK: 5′-adenosine monophosphate-activated protein kinase
ATM: ataxia telangiectasia mutated
BME: basement membrane extract
BrdU: bromodeoxyuridine
CDC25A: dual specificity phosphatase Cdc25A
Cdc37: Hsp90 co-chaperone Cdc37
CDK: cyclin-dependent kinase
CHK2: checkpoint kinase 2
CldU: chlorodeoxyuridine
Cpd2: Compound 2
DDR: DNA-damage response
DDT: DNA-damage tolerance
DMEM: Dulbecco’s modified Eagle’s medium
DSB: DNA double-strand break
EdU: 5-ethynyl-2′-deoxyuridine
FOXM1: Forkhead Box M1
γH2A.X: phospho-histone H2AX
Hsp90: heat shock protein 90
IPA: Ingenuity Pathway Analysis
MCF-7 cells: Michigan Cancer Foundation-7 breast adenocarcinoma cells
MELK: maternal embryonic leucine zipper kinase
p21: cyclin-dependent kinase inhibitor 1
p53: cellular tumour antigen p53
SAMS: substrate peptide for AMPKs
UBA: ubiquitin-associated domain
