# Correction: MELK-T1, a small-molecule inhibitor of protein kinase MELK, decreases DNA-damage tolerance in proliferating cancer cells

**DOI:** 10.1042/BSR-20150194_COR

**Published:** 2018-11-30

**Authors:** Lijs Beke, Cenk Kig, Joannes T. M. Linders, Shannah Boens, An Boeckx, Erika van Heerde, Marc Parade, An De Bondt, Ilse Van den Wyngaert, Tarig Bashir, Souichi Ogata, Lieven Meerpoel, Aleyde Van Eynde, Christopher N. Johnson, Monique Beullens, Dirk Brehmer, Mathieu Bollen

***Bioscience Reports* (2015) 35 (6) e00267; https://doi.org/10.1042/BSR20150194**

By re-analyzing the primary data for the design of follow-up experiments, the authors noted that Figure 4G contained an inadvertent mistake and that the legends of Figures 4F and 4G were inadequately described. Panel 4F shows the average fork-progression rate ± SEM of 619–785 replication forks, with data not from three but from two independent experiments. Panel 4G should have shown the distribution of the fork-progression rate, represented as a percentage of all counted forks in panel 4F. The corrected panel and legends are shown below. These corrections do not affect the description or interpretation of the data. The authors apologize for these errors.

**Figure 4:**
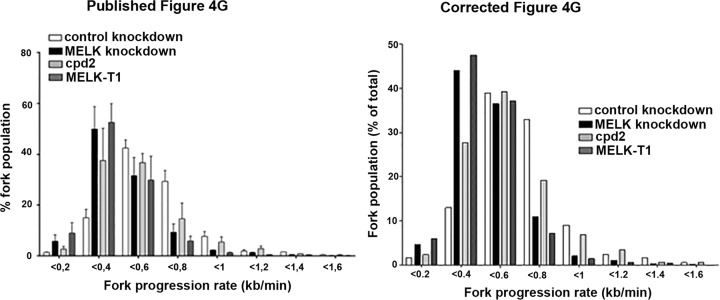
The published and corrected versions of panel G (updated caption for panels F and G below) (**F**) The fork-progression rate was quantified as described previously [25], under the indicated conditions and expressed as kb/min. The results show the mean ± SEM for 619 (control knockdown), 668 (MELK knockdown), 785 (cpd2) and 784 (MELK-T1) replication forks, as derived from two independent experiments. **P<0.001. (**G**) Distribution (% of total) of the progression rates of the forks represented in panel F.

